# Quality of Life Following Dental Rehabilitation with Customized Subperiosteal Implants: A Pilot Cohort Study

**DOI:** 10.3390/medicina62040777

**Published:** 2026-04-16

**Authors:** Evangelos Kostares, Michael Kostares, Georgia Kostare, Fani Pitsigavdaki, Ourania Schoinohoriti, Christos Perisanidis

**Affiliations:** 1 Department of Oral and Maxillofacial Surgery, Dental School, National and Kapodistrian University of Athens, 115 27 Athens, Greece; fpitsi@dent.uoa.gr (F.P.); our.schoino@gmail.com (O.S.); cperis@dent.uoa.gr (C.P.); 2 Department of Otorhinolaryngology—Head and Neck Surgery, “Metaxa” Memorial Anticancer Hospital, 185 37 Piraeus, Greece; michaliskost@med.uoa.gr; 3 Department of Microbiology, Medical School, National and Kapodistrian University of Athens, 115 27 Athens, Greece; gkostare@med.uoa.gr

**Keywords:** dental implants, alveolar bone loss, quality of life, patient satisfaction, prosthesis design, cohort studies

## Abstract

*Background and Objectives*: Severe alveolar atrophy may pose significant challenges for dental rehabilitation. Recent advances in digital planning and CAD/CAM technology have renewed the interest in patient-specific subperiosteal implants as a treatment option for anatomically challenging cases. This cohort study evaluated changes in oral health-related quality of life and patient satisfaction following rehabilitation with customized subperiosteal implants in severe alveolar atrophy. *Materials and Methods*: This cohort study included all consecutive adult patients with severe alveolar atrophy who underwent reconstruction with patient-specific subperiosteal implants at the Department of Oral and Maxillofacial Surgery of “Evangelismos” General Hospital, Athens, Greece, in 2025. Oral health-related quality of life was assessed using the validated OHIP-14 questionnaire preoperatively and 12 months postoperatively. Patient satisfaction was evaluated using a numerical rating scale (NRS). Secondary outcomes included postoperative complications, implant exposure, implant stability, and need for reoperation. Comparisons between baseline and 12-month scores were performed using the Wilcoxon signed-rank test. *Results*: Nine patients who had completed 12-month follow-up were included. Five were male, and all implants were placed in the maxilla. Significant improvement was observed in oral health-related quality of life, with the median OHIP-14 total score decreasing from 41 preoperatively to 1 at the 12-month follow-up. Patient satisfaction also improved significantly, with the median NRS total score increasing from 17 to 58. Improvements were consistent across all OHIP-14 domains and all NRS items. No major complications were recorded. One patient developed early wound dehiscence, and one patient presented with implant exposure at the anterior palate. At the final follow-up twelve months postoperatively, all implants remained clinically and radiographically stable. *Conclusions*: These preliminary short-term findings suggest that customized subperiosteal implants may be a promising option for selected patients with severe alveolar atrophy in whom placement of conventional endosseous implants is not feasible; however, the results should be interpreted cautiously given the very small sample size and observational design.

## 1. Introduction

Alveolar bone atrophy is a common consequence of tooth loss affecting both the maxilla and the mandible. Following extraction or advanced periodontal disease, the absence of functional stimulation initiates a continuous process of bone remodeling characterized by a progressive reduction in alveolar ridge height and width. This resorption occurs in a three-dimensional pattern and may be further accelerated by long-term denture wear or traumatic factors. In the maxilla, bone loss is often more rapid and pronounced due to lower bone density and sinus pneumatization, which further reduces available bone volume. In contrast, mandibular atrophy typically results in ridge narrowing and decreased vertical height, potentially compromising prosthetic stability and implant placement [1,2].

Even with contemporary endosseous osseointegrated implants, placement can be limited by surrounding anatomy, particularly when “gold standard” dimensions are required (≥3.3 mm diameter and ≥8 mm length) and key safety distances must be respected (e.g., ≥1 mm from the maxillary sinus/nasal floor, 2 mm superior to the inferior alveolar canal, 3 mm from adjacent implants, and 1.5 mm from adjacent tooth roots). As resorption progresses, anatomical limitations increasingly complicate rehabilitation, frequently requiring advanced surgical or implant-based approaches. In the maxilla, options such as sinus lifting and regional anchorage with zygomatic or pterygoid implants may facilitate rehabilitation; however, the mandible does not offer comparable regional anchorage, and staged bone augmentation, especially for vertical defects, can be technically challenging and unpredictable. Therefore, understanding the patterns and extent of jaw atrophy is essential for proper treatment planning to restore oral function [1,3,4].

Recent advances in digital planning and computer-aided manufacturing have renewed interest in patient-specific subperiosteal implants for the rehabilitation of severely atrophic jaws. Unlike conventional endosseous implants, which are placed within the alveolar bone, subperiosteal implants are patient-specific titanium frameworks designed to rest directly on the external cortical surface of the jaw, beneath the periosteum, and are stabilized with fixation screws. They usually incorporate transmucosal prosthetic connections that allow subsequent support of a fixed dental prosthesis. In contemporary practice, these implants are manufactured on the basis of CBCT-derived three-dimensional models and CAD/CAM workflows, enabling precise adaptation to the individual bony anatomy. By combining cone beam computed tomography data with CAD/CAM workflows, these implants can be designed to fit the individual bony anatomy precisely and fixed with osteosynthesis screws, potentially improving stability, reducing surgical invasiveness, and avoiding the need for extensive bone augmentation procedures in selected patients. In addition, both subtractive manufacturing (milling) and additive manufacturing (3D printing) have emerged as viable fabrication methods, offering new possibilities for customized treatment in anatomically challenging cases [5,6,7].

Edentulism has been consistently associated with significant deterioration in oral health-related quality of life (OHRQoL), mainly due to impaired mastication, speech difficulties, social stigma, and reduced self-confidence. Conventional complete dentures may partially restore function; however, their effectiveness is highly dependent on stability and retention, and outcomes are often limited in patients with severe ridge atrophy. Implant-based rehabilitation substantially improves patient satisfaction and functional outcomes, with evidence suggesting that restoration of prosthesis stability, rather than prosthesis type alone, is the key determinant of quality-of-life improvement [8,9].

Although patient-specific subperiosteal implants have gained renewed interest due to advances in digital planning and CAD/CAM technologies, the current evidence remains limited, particularly with regard to patient-reported outcomes in individuals with severe alveolar atrophy. In particular, data on oral health-related quality of life and patient satisfaction after rehabilitation with customized subperiosteal implants are still scarce. Therefore, the primary objective of this cohort study was to evaluate changes in oral health–related quality of life following rehabilitation with patient-specific subperiosteal implants in patients presenting with severe alveolar atrophy. Secondary objectives included the assessment of demographic and clinical factors potentially associated with postoperative outcomes. It was hypothesized that reconstruction with customized subperiosteal implants would result in a clinically significant improvement in OHIP-14 scores at twelve months after surgery compared with baseline measurements, accompanied by high postoperative patient satisfaction as assessed by the numerical rating scale (NRS).

## 2. Materials and Methods

### 2.1. Study Design and Setting

Following the STROBE checklist, a prospective cohort study was conducted at the Department of Oral and Maxillofacial Surgery of “Evangelismos” General Hospital, Athens, Greece. All included patients underwent surgery in 2025. All participants were followed for a minimum period of 12 months after surgery. The study evaluated patients undergoing patient-specific subperiosteal implant reconstruction for severe alveolar atrophy. Clinical assessments and data collection were performed at baseline (preoperatively) and during scheduled postoperative follow-up visits, with the primary evaluation conducted twelve months after surgery.

### 2.2. Participants and Eligibility Criteria

Eligible participants were adult patients (>18 years old) diagnosed with severe alveolar atrophy requiring dental rehabilitation with customized subperiosteal implants. Severe alveolar atrophy was defined according to the Cawood and Howell classification (Classes V–VI), based on clinical examination and radiographic assessment using cone-beam computed tomography (CBCT). Participants were consecutively recruited from patients treated at the department during the study period. Inclusion criteria comprised individuals able to provide informed consent and complete patient-reported outcome measures. Patients were excluded if they were unwilling or unable to participate in the postoperative assessments or if they had not completed the final prosthetic restoration.

### 2.3. Surgical Procedure and Follow-Up Protocol

All patients received standardized surgical treatment. The surgical procedure consisted of flap elevation through a crestal approach with releasing incisions, followed by subperiosteal dissection to expose the recipient maxillary bone. The patient-specific titanium subperiosteal framework was then positioned, adapted to the bony surface, and stabilized using fixation screws according to the preoperative design. After connection of the transmucosal prosthetic components, the flaps were trimmed when necessary and closed without tension around the abutments [10].

Postoperative follow-up visits were routinely scheduled at 1 week, 1 month, 6 months, and 12 months after surgery according to departmental clinical protocols. Participants who did not complete the twelve months postoperative evaluation were considered lost to follow-up. Reasons for non-attendance were recorded when available, and outcome assessment was performed using available follow-up data.

All participants completed the planned follow-up period and outcome assessments up to 12 months; therefore, no missing follow-up data were recorded.

### 2.4. Outcomes, Variable and Definitions

The primary outcome of the study was oral health-related quality of life, assessed using the validated OHIP-14 questionnaire [11]. The OHIP-14 was administered preoperatively and repeated twelve months postoperatively. In addition, patient satisfaction was evaluated using an NRS questionnaire. Secondary variables included demographic characteristics (age and sex), clinical parameters related to implant reconstruction and postoperative short-term and long-term outcomes in both short term and long term, including surgical site infection, wound dehiscence, hematoma formation, implant exposure, implant stability and need for reoperation. Surgical site infection was defined according to the CDC guidelines and the surgeon’s clinical judgment. Wound dehiscence was defined as partial separation of the surgical wound margins during healing. Hematoma was considered clinically significant when additional surgical intervention was required. Implant exposure was defined as postoperative exposure of any part of the implant through the overlying mucosa. Implant stability was defined as the absence of clinical mobility and the absence of signs indicating loss of fixation during follow-up. Reoperation was defined as any unplanned secondary surgical intervention related to the implant reconstruction [12,13].

Outcomes, exposures, and potential confounding variables were predefined prior to data collection. Diagnostic and treatment indications were established based on clinical examination and radiographic evaluation using CBCT. Data sources included medical records, surgical reports, and standardized patient questionnaires. All clinical assessments were performed by the same clinical team using uniform evaluation procedures to ensure comparability of measurements across participants. The OHIP-14 and NRS questionnaires were self-completed by patients under supervision to minimize missing data and misinterpretation.

### 2.5. Strategies to Reduce Bias

Several measures were implemented to reduce potential sources of bias. Consecutive patient recruitment minimized selection bias, while standardized surgical techniques and follow-up protocols reduced performance and measurement bias. Outcome assessments were conducted according to a standardized protocol, with consistent application of the same evaluation procedures and scoring criteria at all study time points, in order to minimize potential measurement bias. The use of a validated questionnaire further enhanced the reliability of outcome assessment. Data collection followed predefined protocols, and outcome assessment time points were identical for all participants.

### 2.6. Study Size

The study size was determined by the number of eligible patients treated within the predefined recruitment period. Given the nature of the study, no formal sample size calculation was performed.

### 2.7. Statistical Analysis

All statistical analyses were performed using Stata/BE version 19.0 (StataCorp LLC, College Station, TX, USA). Continuous variables were examined descriptively and are presented using both parametric and non-parametric summary measures, as appropriate. Given the small sample size and the ordinal nature of the patient-reported outcome measures (PROMs), non-parametric methods were prespecified for inferential comparisons.

For the primary analysis of PROMs, results are reported as median and interquartile range (IQR). Domain-level OHIP-14 scores were computed by summing the corresponding item pairs according to the instrument structure, and the total OHIP-14 score was obtained as the sum of all seven domain scores. The numeric rating scale (NRS) total score (range 0–60) was calculated as the sum of the six individual satisfaction items.

Pre- to postoperative changes in OHIP-14 and NRS scores were evaluated using the Wilcoxon signed-rank test for paired samples, which is appropriate for small samples and ordinal paired data. For each comparison, the standardized effect size (r) was calculated as |z|/√n to quantify the magnitude of change independent of sample size [14].

To further characterize the postoperative score distribution and explore potential scale saturation, floor and ceiling effects were evaluated descriptively. The floor effect for the OHIP-14 was defined as the proportion of patients achieving the minimum possible total score (0) at the 12-month follow-up, whereas the ceiling effect for the NRS was defined as the proportion of patients reaching the maximum possible total score (60). Given the exploratory nature of these analyses and the limited sample size, no formal hypothesis testing was applied to floor and ceiling effects. In addition, individual patient trajectories between baseline and 12 months were visualized using line plots to provide a graphical assessment of within-patient change and response consistency.

All statistical tests were two-sided, and a *p*-value < 0.05 was considered statistically significant. Owing to the exploratory design and small cohort size, results were interpreted primarily in conjunction with effect sizes and overall distributional patterns rather than solely on the basis of *p*-values.

### 2.8. Ethical Considerations

The study protocol was approved by the Institutional Review Board/Ethics Committee of “Evangelismos” General Hospital, Athens, Greece (Approval No. 54/18-03-2026). Written informed consent was obtained from all participants prior to inclusion. The study was conducted in accordance with the principles of the Declaration of Helsinki.

## 3. Results

A total of nine patients underwent placement of patient-specific subperiosteal implants. All patients completed the 12-month follow-up and the patient-reported outcome measures. During the postoperative period, no major complications were observed. One patient developed early wound dehiscence that was successfully managed with local flap coverage, while one additional patient presented with implant exposure at the anterior palate. At the final follow-up, all implants remained clinically and radiographically stable. Baseline demographic and clinical characteristics are summarized in Table 1.

Patient-reported outcomes demonstrated an improvement at 12 months. The OHIP-14 total score showed a statistically significant reduction compared with baseline. Improvement was observed across all OHIP-14 domains, with paired comparisons confirming significant postoperative benefit (Table 2).

Similarly, the NRS total score demonstrated a significant increase at the 12-month follow-up, indicating improved patient satisfaction. All individual NRS items (aesthetics, chewing, comfort, phonetics, cleaning, and general satisfaction) showed statistically significant improvement (Table 3).

**Figure 1:**
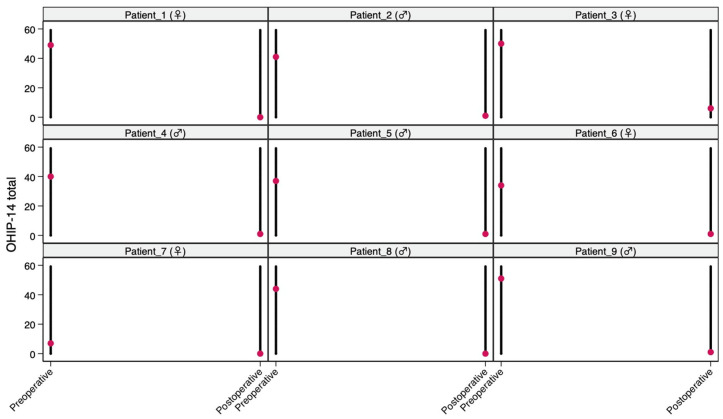
Individual OHIP-14 total trajectories from baseline to 12 months (spaghetti plot).

**Figure 2:**
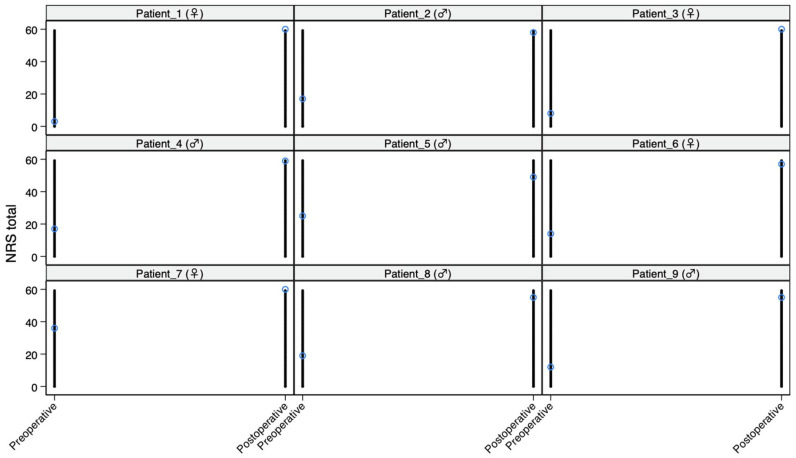
Individual NRS total trajectories from baseline to 12 months (spaghetti plot).

Individual trajectory plots illustrating pre- to postoperative changes are presented in Figure 1 (OHIP-14) and Figure 2 (NRS). Descriptively, 3/9 patients (33.3%) reached the minimum postoperative OHIP-14 total score and 3/9 patients (33.3%) reached the maximum postoperative NRS total score (Table 4).

Median change represents postoperative minus preoperative paired difference. Bootstrap percentile 95% CIs were obtained from 5000 resamples. Floor and ceiling effects denote the proportion achieving the scale minimum or maximum at 12 months.

## 4. Discussion

The principal aim of subperiosteal implants is to achieve prosthetic dental rehabilitation in patients with severe alveolar or jaw atrophy for whom conventional endosseous implant therapy is not feasible or predictable. The recent consensus report on customized subperiosteal implants emphasizes that this treatment modality is intended not only to replace missing dentition, but also to restore essential oral functions such as mastication, phonation, and swallowing, while improving prosthetic stability, aesthetics, and oral health-related quality of life. In addition, the report notes that immediate loading protocols may facilitate earlier functional rehabilitation and minimize disruption to patients’ social and occupational lives [15,16].

From a clinical decision-making perspective, customized subperiosteal implants should be considered mainly in carefully selected patients with severe jaw atrophy, especially Cawood–Howell class V–VI cases, when conventional endosseous implants cannot be placed in a predictable manner and when extensive regenerative procedures are either contraindicated, declined by the patient, or associated with excessive morbidity. Current consensus recommendations suggest that these implants are particularly useful in edentulous patients seeking fixed rehabilitation, including cases with previous graft failure, intolerance to removable prostheses, or medical/anatomical conditions that limit staged augmentation [17]. In comparison, zygomatic implants are primarily indicated for the severely atrophic maxilla when residual zygomatic bone allows anchorage and when a graftless fixed solution is preferred; however, they require advanced surgical expertise and are associated with specific complications, most notably maxillary sinusitis, as well as oroantral communication, soft-tissue problems, peri-implant infection, and, more rarely, orbital or other anatomical complications [18]. Therefore, the main comparative advantage of customized subperiosteal implants is the possibility of individualized, graft-avoidant full-arch rehabilitation with reduced treatment burden in anatomically complex cases, whereas their disadvantages include the need for meticulous digital planning, dependence on soft-tissue quality, limited long-term evidence, and the fact that indications should remain restricted to highly selected patients rather than routine implant candidates [17,18].

Available evidence indicates that patient-specific subperiosteal implants can achieve encouraging survival and acceptable medium- to long-term clinical performance in carefully selected patients with severe jaw atrophy. In the recent retrospective study by Borre et al. (2025) [19], 40 customized mandibular subperiosteal implants placed in 19 patients demonstrated an implant survival rate of 92.5% at a mean follow-up of 804 days (approximately 2.2 years), with high patient satisfaction despite mucosal recession in nearly one-third of implants. In addition, Loginoff et al. (2025) [20] reported that, among 10 custom-made maxillary subperiosteal implants followed for 1 to 10 years, 8 implants remained functionally and clinically stable, corresponding to a 10-year Kaplan–Meier survival probability of 80%. At the same time, the available literature remains constrained by small sample sizes, retrospective designs, and heterogeneous follow-up periods, with many studies still limited to 1–5 years of observation. Therefore, while current data support the clinical viability of patient-specific subperiosteal implants and suggest that favorable outcomes may be maintained beyond the short term, the true long-term predictability of these devices still requires confirmation in larger prospective cohorts with standardized outcome reporting.

In addition to survival, previous studies have also highlighted the positive effect of these implants on oral health-related quality of life Borre et al. (2022) [21], demonstrated significant improvement in OHRQoL following placement of additively manufactured subperiosteal jaw implants, with OHIP-14 scores markedly decreasing over 12 months and high patient satisfaction achieved without major complications. These findings support the concept that restoring stable fixed rehabilitation in anatomically compromised patients can lead to meaningful patient-reported benefits, aligning with the improvements observed in the present study. Similarly, in a subsequent study Borre et al. (2025) [19], reported favorable outcomes using additively manufactured subperiosteal implants in patients with severe mandibular atrophy, including 19 patients (14 females and 5 males) treated with 40 customized implants and followed for a mean of 804 days. The implant survival rate was 92.5%, while patient-reported outcomes demonstrated high satisfaction, reflected by a low mean OHIP-14 score of 6.68 and high overall NRS scores. Although mucosal recession was observed in approximately one-third of the implants, it did not negatively affect patient perception or implant function. Notably, a thin soft-tissue biotype was identified as a significant risk factor for peri-implant recession, highlighting the importance of careful soft-tissue assessment during treatment planning.

In the present small observational cohort, dental rehabilitation with patient-specific subperiosteal implants was associated with in a substantial improvement of patient-reported outcomes at the 12-month follow-up, as is reflected by the marked reduction in OHIP-14 scores and the parallel increase in NRS satisfaction scores. These preliminary findings suggest that this treatment modality may effectively restore not only oral function, but also psychosocial well-being of patients, suffering from severe alveolar atrophy. The consistency of improvement across all OHIP-14 domains and all satisfaction items further supports the considerable impact of treatment on the patients’ daily life. Moreover, the favorable complication profile and maintenance of implant stability in all cases indicate that these patient-reported benefits were achieved along with encouraging short-term clinical outcomes.

This study has several methodological strengths. Consecutive patient recruitment, uniform surgical protocols, and predefined outcome assessment time points reduced potential selection and performance bias. The use of validated patient-reported outcome measures (OHIP-14 and NRS) enhanced the clinical relevance and reliability of the findings, while evaluation by the same clinical team ensured methodological consistency across participants. Nevertheless, important limitations should be considered. The very small sample size (*n* = 9) substantially limited statistical power, reduced the precision of the estimates and the generalizability of the findings, and precluded the detection of clinically meaningful effects as well as subgroup analyses. Therefore, the present results should be considered highly preliminary and hypothesis-generating rather than conclusive. In addition, although the use of non-parametric methods, albeit appropriate for ordinal data and small cohorts, restricted more detailed modeling of predictors of outcome. The single-center design and recruitment from a single geographical region may limit external validity, as patient characteristics, surgical expertise, and healthcare system factors may differ across institutions and countries. The 12-month follow-up period provides meaningful short-term outcomes but does not allow assessment of long-term implant performance, biological complications, or stability of patient-reported benefits. Postoperative clinical assessments were performed by the same treating team, so observer bias cannot be completely excluded. Because the study had no control group, residual confounding cannot be ruled out, and some of the observed improvement may have been influenced by factors other than the intervention itself. Finally, the descriptive postoperative clustering of scores at the lower end of the OHIP-14 scale and the upper end of the NRS suggests possible scale saturation, which may reduce sensitivity for detecting subtle differences among patients with highly favorable outcomes [22,23].

## 5. Conclusions

In conclusion, dental rehabilitation with patient-specific subperiosteal implants in patients with severe alveolar atrophy was associated with substantial improvement in oral health-related quality of life and high levels of patient satisfaction at 12 months postoperatively, together with favorable short-term clinical outcomes. These findings suggest that customized subperiosteal implants may represent a valuable treatment option for carefully selected patients in whom conventional endosseous implant placement is limited or not feasible. Nevertheless, the results should be interpreted with caution given the small sample size, single-center design, and relatively short follow-up period. Further prospective studies with larger cohorts, longer observation periods, and multicenter designs are needed to confirm these findings, better define complication patterns and risk factors, and clarify the long-term stability of both clinical and patient-reported outcomes.

## Figures and Tables

**Table 1:** Baseline characteristics and postoperative outcomes (case-level).

Patient	Gender	Age	Site	Complications	Status
1	Male	68	Maxilla	Wound dehiscence	Stable
2	Male	64	Maxilla	None	Stable
3	Female	62	Maxilla	None	Stable
4	Male	73	Maxilla	None	Stable
5	Male	68	Maxilla	None	Stable
6	Male	72	Maxilla	None	Stable
7	Female	74	Maxilla	None	Stable
8	Female	73	Maxilla	None	Stable
9	Female	68	Maxilla	Implant exposure	Stable

**Table 2:** OHIP-14 total and domain scores at baseline and 12 months (*n* = 9).

Outcome	Baseline (Median [IQR])	12 Months (Median [IQR])	Wilcoxon z	*p* (Asymptotic)	Effect Size
OHIP-14 total	41 [37–49]	1 [0–1]	2.668	0.0076	0.889
Domain 1	6 [4–6]	0 [0–1]	2.570	0.0102	0.857
Domain 2	7 [4–8]	0 [0–0]	2.680	0.0074	0.893
Domain 3	7 [3–8]	0 [0–0]	2.692	0.0071	0.897
Domain 4	7 [7–7]	0 [0–0]	2.694	0.0071	0.898
Domain 5	6 [5–7]	0 [0–0]	2.618	0.0088	0.873
Domain 6	5 [5–8]	0 [0–0]	2.528	0.0115	0.843
Domain 7	7 [5–8]	0 [0–0]	2.622	0.0087	0.874

**Table 3:** NRS total and item scores at baseline and 12 months (*n* = 9).

Outcome	Baseline (Median [IQR])	12 Months (Median [IQR])	Wilcoxon z	*p* (Asymptotic)	Effect Size
**NRS total (0–60)**	17 [12–19]	58 [55–60]	−2.670	0.0076	0.890
**Aesthetics**	2 [1–4]	9 [9–10]	−2.618	0.0088	0.873
**Chewing**	1 [0–2]	10 [9–10]	−2.692	0.0071	0.897
**Comfort**	1 [1–4]	10 [10–10]	−2.670	0.0076	0.890
**Phonetics**	3 [1–5]	10 [9–10]	−2.670	0.0076	0.890
**Cleaning**	4 [1–6]	10 [9–10]	−2.680	0.0074	0.893
**General satisfaction**	2 [1–4]	10 [10–10]	−2.684	0.0073	0.895

**Table 4:** Distributional effects and bootstrap confidence intervals for median change (postoperative vs. preoperative), *n* = 9.

Metric	Estimate
**OHIP-14**	
Postoperative floor (score = 0)	3/9 (33.3%)
Median change in total score (postoperative–preoperative)	−40
Bootstrap percentile 95% CI	[−49, −33]
**NRS**	
Postoperative ceiling (score = 60)	3/9 (33.3%)
Median change in total score (postoperative–preoperative)	42
Bootstrap percentile 95% CI	[24, 52]

## Data Availability

The data supporting the findings of this study are available within the article. Additional data are not publicly available due to privacy and ethical restrictions.
